# Detection of humoral and cellular immune response to anti-SARS-CoV-2 BNT162b2 vaccine in breastfeeding women and naïve and previously infected individuals

**DOI:** 10.1038/s41598-023-33516-1

**Published:** 2023-04-17

**Authors:** Milena Cavic, Andrijana Nesic, Katarina Mirjacic Martinovic, Ana Vuletic, Irina Besu Zizak, Nevena Tisma Miletic, Ana Krivokuca, Radmila Jankovic, Marija Gavrovic-Jankulovic

**Affiliations:** 1 Department of Experimental Oncology, Institute for Oncology and Radiology of Serbia, Pasterova 14, 11000 Belgrade, Serbia; 2 Department of Biochemistry, Faculty of Chemistry, University of Belgrade, Belgrade, Serbia

**Keywords:** Biochemistry, Immunology, Diseases

## Abstract

This study explored humoral and cellular responses to anti-SARS-CoV-2 BNT162b2 mRNA vaccine in breastfeeding women and naïve and seropositive individuals in the first six months after vaccination.Sixty-one volunteers vaccinated with two doses of the BNT162b2 mRNA vaccine were enrolled in the study. In-house developed ELISA was used for the quantification of SARS-CoV-2 RBD-specific antibodies. Cell surface marker expression and intracellular IFN-γ analysis were carried out by flow cytometry. The concentrations of IFN-γ, IL-6 and TNF were determined by ELISA. A significant rise in anti-RBD IgG antibody levels was observed 14 days after the first vaccine dose (p < 0.0001) in serum and milk. The expression of CD28 on CD4^+^ T cells was significantly higher compared to baseline (p < 0.05). There was a significant increase (p ≤ 0.05) in B cell lymphocyte subset after revaccination, and increased percentage of CD80^+^ B cells. The expression of IFN-γ in peripheral blood lymphocytes, CD3^+^ T cells and serum was significantly increased (p < 0.05). No significant difference in immune response was observed between breastfeeding women and other study participants. The anti-SARS-CoV-2 BNT162b2 mRNA vaccine-induced measurable and durable immune response in breastfeeding women and in naïve and previously infected individuals.

## 1. Introduction

The global widespread of the SARS-CoV-2 virus after the first official case was registered on Dec 31, 2019 in Wuhan, China, led to a worldwide pandemic of the C*oronavirus Disease-19* (COVID-19), with aroun 648 million cases and 6.6 million deaths until Dec 18, 2022^1^. Mortality in COVID-19 has been associated with excessive production of proinflammatory cytokines such as IL-6, TNF-α, IL-1, the so-called "cytokine storm", which may lead to acute respiratory distress syndrome and extensive tissue damage resulting in multi-organ failure^2^. However, rapid improvements in diagnostics, treatment, and the introduction of vaccination were expected to contribute to a steady decline in the number of infected individuals, the need for hospitalization, and ultimately a reduction in mortality rate. In Serbia, the first officially registered case occurred on Mar 6, 2020, and vaccination was initiated on Dec 24, 2020. Currently, there are four vaccines available to the general population, Vero Cell–Inactivated (Sinopharm), Vaxzevria (ChAdOx1-S [recombinant], AstraZeneca), Sputnik V (Gam-COVID-Vac, Gamaleya), and Comirnaty (BNT162b2, BioNTech–Pfizer).

In addition to the production of specific antibodies, effective immune response against SARS-CoV-2 requires the activation of cellular immunity. The components of cellular immunity, including helper CD4^+^ and cytotoxic CD8^+^ T cells, natural killer (NK) cells, and B cells, within the first hours and days after exposure to this virus are expected to reduce disease symptoms, preventing disease progression, hospitalization, and death. Activated T-helper (Th) cells can indirectly kill infected cells, support B cell function and antibody response, by producing interferon-γ (IFN-γ)^3,4^. It has been shown that Th1 phenotype is associated with lower severity of COVID-19 disease^5^, thus strategies for the development of COVID-19 vaccines should also consider the possibility of activation Th cells for IFN-γ production. Cellular immunity to SARS-CoV-2 is reported to be detectable up to 6 months post-infection in over 90% of individuals^6^. Thus, it was expected that vaccination would give rise to a measurable cellular immunity that would contribute to the battle against the pandemic. New results have shown that besides activating B cells in germinal centers^7^, anti-SARS-CoV-2 mRNA vaccines should generate spike protein-specific CD4^+^ and CD8^+^ T cell responses^8^. Although antibody titer usually correlates with vaccine efficacy, B and T cells are important in protection upon re-exposure to virus antigens. These memory B and T cells are expected to be generated by SARS-CoV-2 mRNA vaccines and should have a role in controlling the replication of the virus, limiting viral dissemination^9^.

Although no severe adverse events have been reported, the specific response to anti-SARS-CoV-2 vaccination in pregnant and breastfeeding women and infants whose mothers received the vaccine during lactation is still under evaluation and remains a controversial topic^10,11^. This research aimed to explore for the first time the monthly humoral and cellular responses to anti-SARS-CoV-2 BNT162b2 mRNA vaccine in breastfeeding women and in naïve and seropositive individuals, in the first six months after vaccination in Serbia, and a two-year self-reported follow-up of long-term safety.

## Material and methods

### Study design and sample collection and processing

A total of 61 volunteers (45 SARS-CoV-2 naive, 16 SARS-CoV-2 recovered according to PCR-confirmed reports) were enrolled in the study and monitored in the period from January to November 2021. The average time between natural infection and vaccination in previously infected individuals was 3.18 ± 0.98 months, with a range of 2–5 months. All participants were older than 18 years of age and showed no signs of respiratory infections at the time of vaccination. Most participants were healthcare workers from the Institute for Oncology and Radiology of Serbia (44; 72%). Participants were vaccinated with two doses of the BNT162b2 mRNA vaccine (3 weeks apart). All blood draws were performed in approved medical centers under strict protocols and observation. Four participants were breastfeeding mothers who volunteered matching blood and milk samples. Samples were collected at 6-time points: point 1—pre-vaccination (baseline), point 2–14 days post-primary immunization, point 3–1 month post-primary immunization, point 4–2 months post-primary immunization, point 5–3 months post-primary immunization, and point 6–6 months post-primary immunization. 10–20 mL of peripheral blood was sampled for each time point, as well as 1 mL of milk for 4 participating breastfeeding women. Serum was obtained from peripheral blood by centrifugation immediately upon blood draw and stored at −20 °C. Peripheral blood mononuclear cells (PBMC) were isolated from heparinized blood using Lymphoprep (Nypacon, Oslo, Norway) density gradient, centrifuged at 500 g for 40 min, and washed three times in RPMI 1640 culture medium (CM) (Sigma, St. Louis, MO) supplemented with 10% fetal calf serum (FCS) (Sigma, St. Louis, MO). After washing, PBMC were immediately used for immunophenotypic analysis.

The study was approved by the Ethics Committee of the Institute for Oncology and Radiology of Serbia (No. 899–01 from 01.04.2021.), and all participants signed an informed consent. All participants were interviewed by experienced medical staff. Long term self-reported follow-up of post-vaccination adverse effects was conducted during two years after the first vaccine dose.

### Detection and quantification of SARS-CoV-2 RBD-specific antibodies

Expression of RBD of SARS-CoV-2 spike protein (NCBI: YP_009724390, AA319-541) was carried out in the HEK-293 T cell line following a previously published procedure^12^. The recombinant protein was purified until homogeneity, and the yield obtained was 500 mg per L of the cell culture. Briefly, the recombinant RBD protein was used to coat flat-bottom 96-well plates (Thermo Scientific NUNC-MaxiSorp, USA) at a final concentration of 10 μg/mL (50 μL/well) in coating buffer (15 mM Na_2_CO_3_/35 mM NaHCO_3_, pH 9.5) at 4 °C overnight. The plates were washed with TBS containing 0.05% Tween 20 (TBST) and blocked with 1% BSA in TBST for 2 h at 37 °C. Diluted sera were incubated at 37 °C for 1 h in TBST containing 0.1% BSA, and then the plates were washed with TBST. For total specific IgG determination, goat anti-Human-IgG-AP conjugated antibody (Novus Biologicals, USA) was diluted 1/1000 in blocking solution and added to the wells. After incubation for 1 h at 37 °C, plates were washed 5 times with TBST and developed with 4-Nitrophenyl phosphate for 1 h at 37 °C. The absorbance was measured at 450 nm (A_450_).

### Flow cytometric analysis

Cell surface immunophenotype of peripheral blood mononuclear cells (PBMCs) was identified using the following combinations of directly labeled monoclonal antibodies (mAbs): CD3FITC/CD28PE/CD4PerCP, CD80PE/CD19PerCP, CD80PE/CD14PerCP (Becton Dickinson, San Jose, USA). The samples were prepared as previously described^13^ using 1 × 10^5^ freshly isolated PBMCs in 100 μL RPMI 1640 supplemented with 10% Fetal Calf Serum (FCS, Sigma)*,* incubated for 30 min at 4 °C with 20 μL of appropriate mAb combination, washed twice with ice-cold phosphate-buffered saline (PBS), and fixed with 1% paraformaldehyde before FACS analyses. Surface marker expression was quantified on FACSCalibur flow cytometer (Becton–Dickinson, San Jose, USA). A total of 10,000–50,000 gated events verified as peripheral blood lymphocytes (PBLs) and monocytes, according to their physical characteristics, forward scatter characteristics (FSC), and side scatter characteristics (SSC), were collected per sample and analyzed using CellQUEST software. The cell subsets were expressed as the percentage in peripheral blood.

### Intracellular IFN-γ analysis

For intracellular staining of IFNγ 1 × 10^6^ PBMCs were incubated with Phorbol 12*-*myristate 13*-*acetate (PMA) (50 ng/mL) plus ionomycin (500 ng/mL) for 4 h at 37 °C and with Brefeldin A (10 μg/mL) for the last 3 h, as previously described^14^. Cells were first stained for surface antigen with CD3 PerCP antibody, fixed and after permeabilization with BD FACS permeabilizing solution 2 (BD Biosciences) stained for anti-IFN-γ PE (Becton Dickinson). Intracellular IFNγ is expressed as the percentage in PBLs, as well as in CD3 T lymphocytes.

### ELISA

The concentrations of IFN-γ, IL-6, and TNF in the sera of the investigated participants were determined by commercial uncoated ELISA kits, according to manufacturer instructions (Invitrogen). The concentrations of cytokines were determined at points 1 (before vaccination), point 3 (1-month post-primary immunization), and point 4 (2 months post-primary immunization). Briefly, ELISA plates were coated with appropriate capture antibodies in coating buffer and incubated overnight at 4 °C. First, the plates were washed and blocked with ELISA/ELISASPOT Diluent. After 1 h incubation, the plates were washed, and serum samples and standards were added to the wells. After incubation, washing, and aspiration, according to the manufacturer's instructions, the detection antibody solution was added to each well. Subsequently, the plates were washed and aspirated; the avidin-HRP solution was added to the wells and incubated for 30 min. Next, the plates were washed, and (3,3′,5,5′-Tetramethylbenzidine) TMB solution was added to each well. The plates were incubated for 15 min at room temperature, and the enzymatic reaction was terminated by adding a stop solution. The absorbance was measured at a 450/520 nm using the Multiskan EX Thermo Labsystems plate reader^15^.

### Statistical analysis

The results /are presented as individual values with mean ± standard error of the mean (SEM). Differences between values were compared using unpaired t-test and Wilcoxon signed-rank test and the results were considered statistically significant if p < 0.05. The analysis was performed using GraphPad Prism 9.0 (GraphPad, La Jolla, CA, USA).

## Results

### 3.1 Study participants and serum/milk processing

Characteristics of study participants are presented in Table 1. From 61 volunteers who received the anti-SARS-CoV-2 BNT162b2 mRNA vaccine, a total of 344 serum samples were collected at 6 time points, starting from pre-vaccination baseline to 6 months post-first vaccination point. Most study participants (97%) reported local adverse reactions to vaccination in the form of mild arm/shoulder pain and fatigue, which lasted 1–2 days. Only three participants received the second vaccine dose with delay, two due to adverse effects upon primary vaccination (skin rash, allergy), and one due to a finding of pregnancy after the first dose. No infection was reported between two vaccine doses. After six months of monitoring, only 3 (5%) of participants were infected with the SARS-CoV-2, and no serious form of illness that required hospitalization was detected. Seven participants got pregnant after vaccination, one 2 weeks after the first dose, and one 4 months after the first dose, and 5 over 12 months after the first dose. Long term self-reported follow-up revealed no vaccination-related adverse effects up to two years after the first dose, in breastfeeding women or any other study participant.

**Table 1:** Characteristics of study participants.

Characteristics	N (%)
Gender	
Male	10 (16.4)
Female	51 (83.6)
Age (years)	
Range	20–62
Median	40
Smoking status	
Non-smoker or ex-smoker ≥ 30 years	30 (49.1)
Smoker or ex-smoker < 30 years	31 (50.9)
Pre-vaccination SARS-CoV-2 infection	
Yes	14 (22.9)
No	47 (77.1)

### Quantification of SARS-CoV-2 RBD-specific antibodies in the serum and milk of study participants

**Figure 1:**
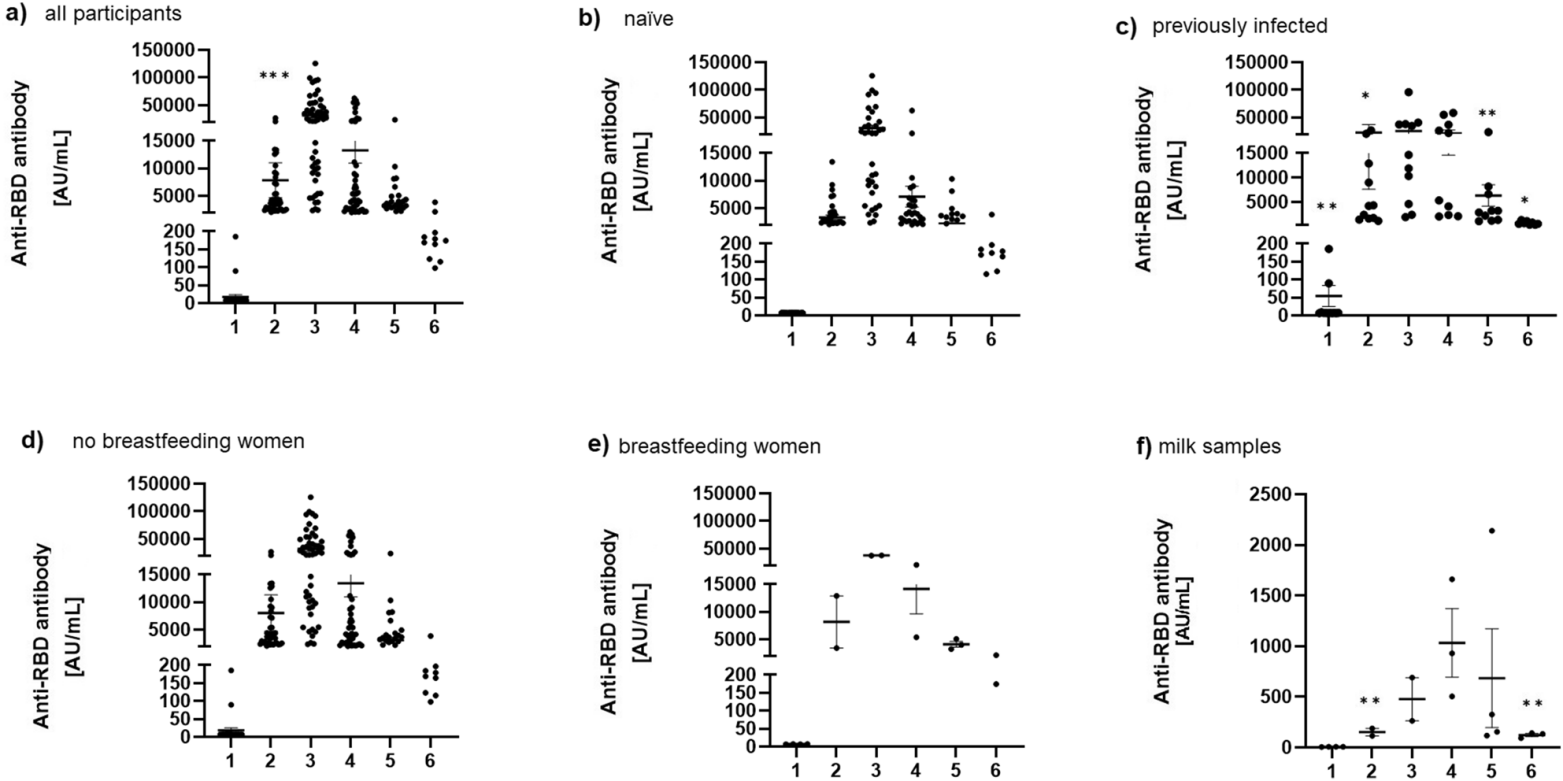
Serum anti-RBD IgG antibody levels in the (**a**) whole analyzed group, (**b** naïve, **c**) previously infected individuals, (**d**) whole group without breastfeeding women, (**e**) breastfeeding women and (**f**) in the milk of breastfeeding women. The results are presented as individual values with mean ± standard error of the mean (SEM) of three independent replicates. Differences between values were compared using unpaired t-test and the results were considered statistically significant if *p* < 0.05. ^*^p < 0.05; ^**^p < 0.01; ^***^ *p* < 0.0001.

Analyzing the levels of post-vaccination anti-RBD IgG antibodies in serum, a significant rise in anti-RBD IgG levels was observed even 14 days after the first vaccine dose in the whole analyzed group compared to IgG levels prior to vaccination (17.47 ± 51.49 AU/mL vs. 7828.28 ± 24,157.29 AU/mL, p < 0.0001) (Fig. 1a). The serum levels of anti-RBD IgG continued to rise up to 2 months post-vaccination, when a decrease was detected, which lasted up to 6 months, steadily. The levels of anti-RBD IgG antibodies were compared at each time point between naïve and previously infected individuals (Fig. 1b-c), and it was determined that the difference was statistically significant at baseline (point 1), as previously infected individuals had a higher level of antibodies prior to vaccination (7.23 ± 0.77 AU/mL vs. 54.47 ± 101.40 AU/mL, p < 0.01). The difference was statistically significant also in points 2 (3979.48 ± 3277.27 AU/mL vs. 22,581.99 ± 49,570.63 AU/mL, p < 0.05), 5 (2369.16 ± 1934.31 AU/mL vs. 6366.83 ± 6875.85 AU/mL, p < 0.01), and 6 (461.96 ± 375.41 AU/mL vs. 766.04 ± 317.35 AU/mL, p < 0.05). The decline in antibody levels had the same dynamics in both groups but remained at higher levels in previously infected individuals up to 6 months. No other analyzed parameters were correlated with a significantly different level of antibodies in serum (age, gender, smoking status). When comparing levels of anti-RBD IgG antibodies in other participants and breastfeeding women (Fig. 1d-e), no significant differences in serum antibody levels were observed in any of the comparisons. Anti-RBD IgG antibodies were also detected in the milk of 4 breastfeeding women (Fig. 1f). In lower levels, they presented with the same dynamics of rising and decline as detected in the serum. Comparing the levels of anti-RBD IgG antibodies in milk at each time point to baseline, (Fig. 1f), it was determined that the difference was statistically significant at points 2 (6.57 ± 0.77 AU/mL vs. 152.13 ± 35.83 AU/mL, p < 0.01) and 6 (6.57 ± 0.77 AU/mL vs. 117.31 ± 25.64 AU/mL, p < 0.01).

### Flow cytometric and intracellular IFN-γ analyses

**Figure 2:**
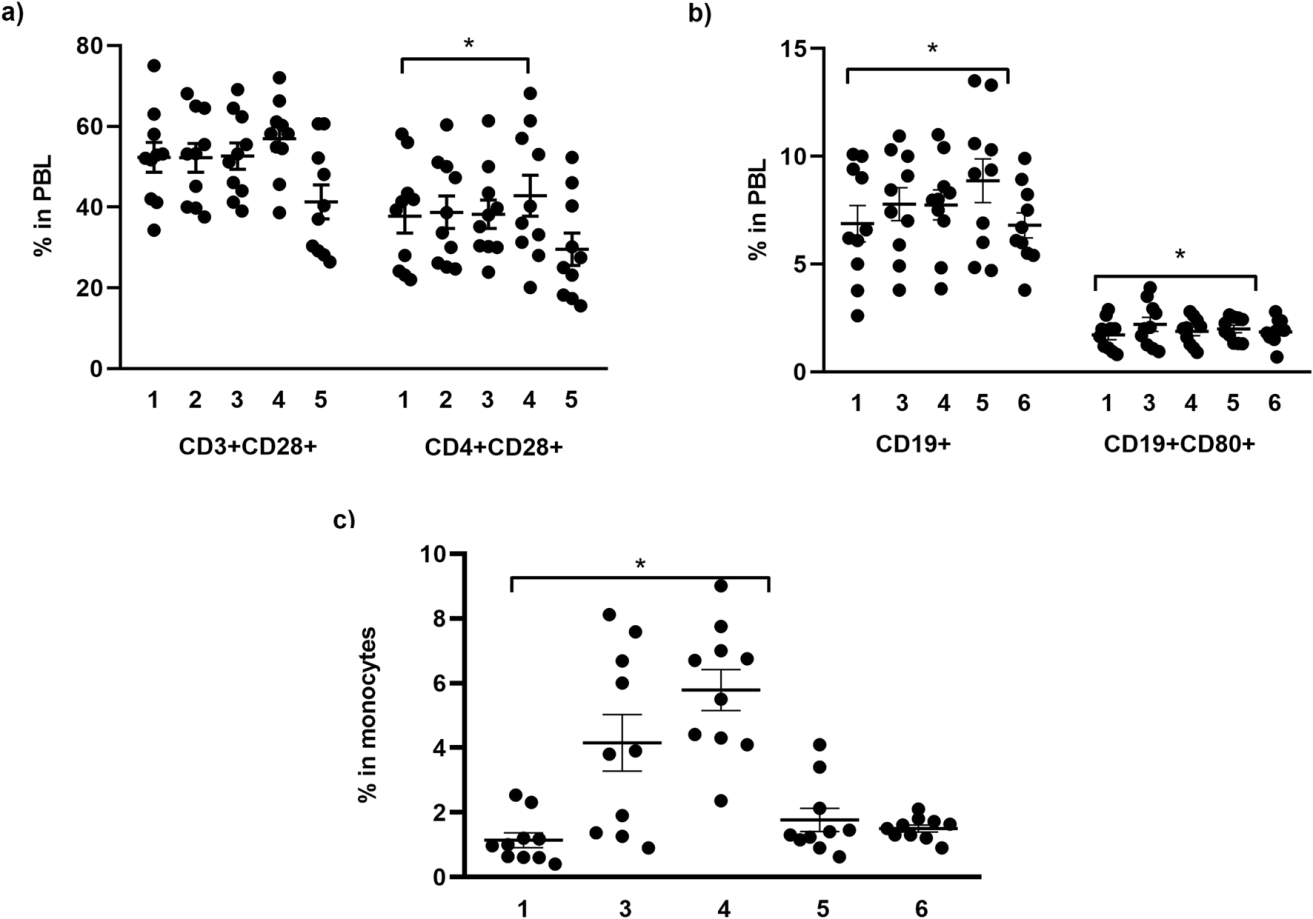
Percentage of (**a**) CD3^+^CD28^+^ and CD4^+^CD28^+^ T lymphocytes, (**b**) CD19^+^ and CD19^+^CD80^+^ B lymphocytes, (**c**) CD14^+^CD80^+^ monocytes in peripheral blood. The results are presented as individual values with mean ± standard error of the mean (SEM). Differences between values were compared using Wilcoxon signed-rank test and the results were considered statistically significant if *p* < 0.05. ^*^p < 0.05.

Data obtained by flow cytometric analysis showed that the expression of CD28 on CD4^+^ T cells in peripheral blood of at 2 months after the second vaccine dose was significantly higher compared to the values before vaccination (42.84** ± **5.37% vs. 37.75 ± 5.96%, p < 0.05, Wilcoxon signed-rank test) (Fig. 2a).

There was a significant increase (p ≤ 0.05, Wilcoxon signed rank test) in the prevalence of B cell lymphocyte subset (CD19^+^) from 6.87 ± 1.17% to 8.73 ± 1.54%, two months after revaccination, as well as an increased percentage of CD80^+^ B cells from 1.72 ± 0.28% to 1.99 ± 0.28% (Fig. 2b). Furthermore, five weeks after revaccination, a significant (p ≤ 0.05, Wilcoxon signed rank test) increase in the percentage of CD14^+^CD80^+^ cells from 1.15 ± 0.38% to 5.87 ± 0.95% was detected in the monocyte gate (Fig. 2c).

**Figure 3:**
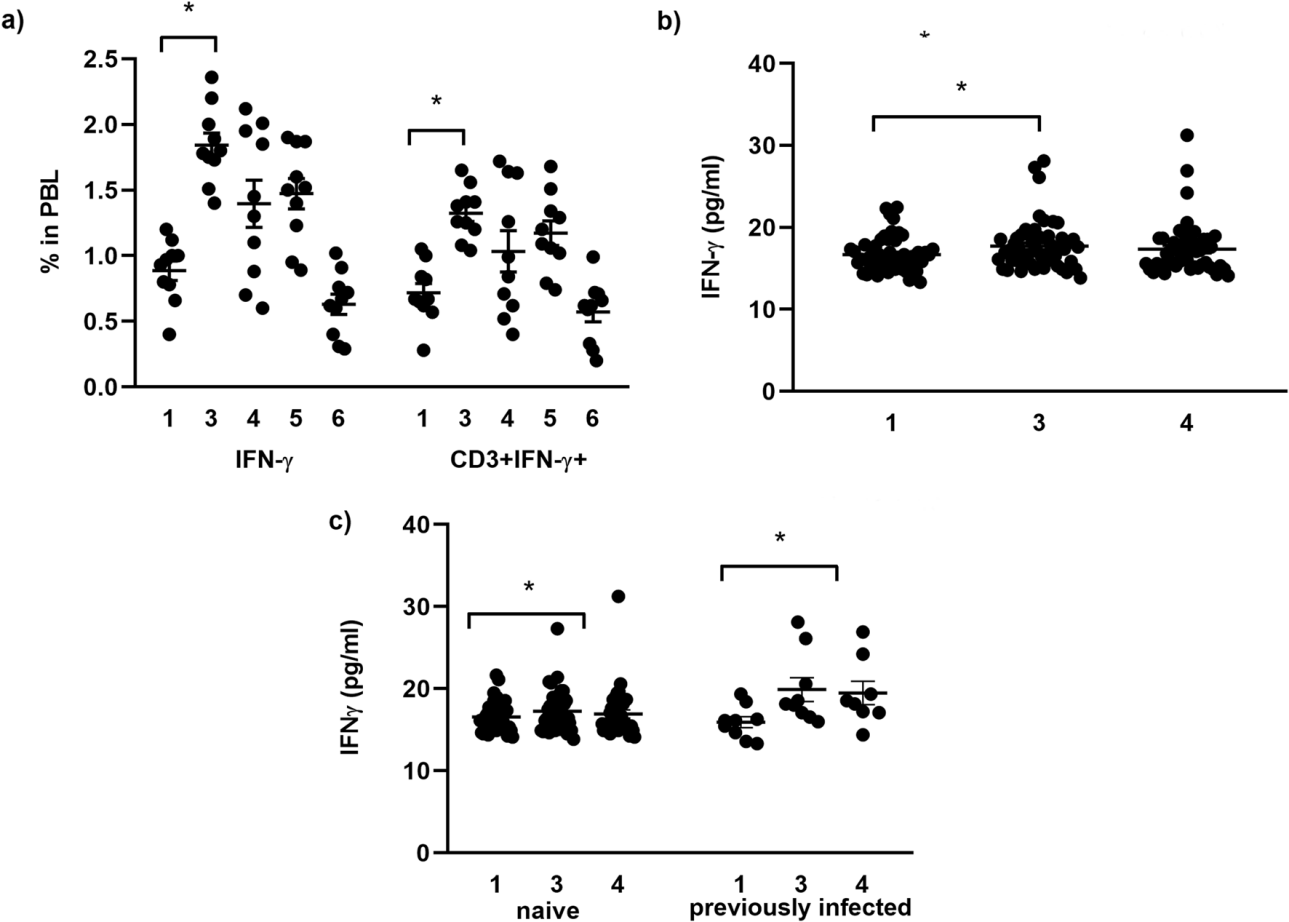
(**a**) Expression of IFN-γ in all and CD3^+^ T peripheral blood lymphocytes. Serum level of IFN-γ in the (**b**) whole analyzed group, (**c**) naïve and previously infected individuals. The results are presented as individual values with mean ± standard error of the mean (SEM). Differences between values were compared using Wilcoxon signed-rank test and the results were considered statistically significant if *p* < 0.05. ^*^p < 0.05.

The expression of IFN-γ in PBLs, as well as in CD3^+^ T cells was significantly (p < 0.05, Wilcoxon signed rank test) increased at 1 week after the second vaccine dose (1.84** ± **0.13% in PBL and 1.32** ± **0.10% in CD3^+^ T cells) compared to pre-vaccination baseline values (0.88** ± **0.13% in PBL and 0.72** ± **0.12% in CD3^+^ T cells) (Fig. 3a). Furthermore, serum levels of IFN-γ were significantly increased in all participants from the initial value of 16.69 ± 0.29 pg/mL to 17.72 ± 0.41 pg/mL (p < 0.05, Wilcoxon signed rank test) (Fig. 3b), as well as in naive from 16.55 ± 1.77 pg/mL to 17.26 ± 2.53 pg/mL and in a group and previously infected individuals, from 15.93 ± 2.01 pg/mL to 19.90 ± 4.32 pg/mL at 1 week after revaccination (Fig. 3c).

There was no significant (p > 0.05, Wilcoxon signed rank test) difference in the serum levels of IL-6 and TNF-α 1 week and five weeks after the second vaccine dose compared to pre-vaccination values in the whole group, as well as in naive or previously infected individuals (Supplementary Fig. 1a–d).

## Discussion

The SARS-CoV-2 respiratory infection outbreak had a profound negative effect on various aspects of everyday life, including societal, educational, and health-related issues not in direct connection with the COVID-19 disease itself^16–18^. A global vaccination approach was necessary to complement distancing measures in combating the disease, and many studies explored the efficiency and effectiveness of various vaccine types distributed worldwide during 2021. In Serbia, only interim reports on humoral immunity after 2 and 3 months after vaccination are currently available^19,20^ and are in accordance with data reported in this study. In our analyzed cohort of up to 6 months upon vaccination, a 100% seroconversion rate was detected after the first vaccine dose. Only 5% of participants had to receive the second vaccine dose with delay. There were no reports of SARS-CoV-2 infection between two vaccine doses. Up to six months of monitoring, only 5% of participants were infected, without the need for hospitalization. Titers of anti-RBD SARS-CoV-2 IgG antibodies were significantly higher compared to baseline even after the first vaccine dose, which contributes to the notion that using the BNT162b2 mRNA vaccine is an efficient approach for fast humoral responses against the original virus strains. The IgG serum levels continued to rise up to 2 months post-vaccination, when a steady decrease was detected up to the analyzed period of 6 months. Previously infected individuals were found to have higher IgG titers compared to naïve individuals up to 6 months, which was the only factor contributing to different antibody levels. Anti-RBD IgG antibodies were also detected in the milk of breastfeeding women in our and some previous studies^21^, with matching titer dynamics as in the serum. Similar reports have been published for IgA antibodies after natural infection^22^. As limited data are currently available on the risks and benefits of vaccination of breastfeeding women, these results are promising in the light of a possible passive immunization of breastmilk-fed infants, even after 6 months upon vaccination of mothers. No significant differences in serum antibody levels were observed between naïve or previously infected individuals and breastfeeding women, reinforcing observations that the response to anti-SARS-CoV-2 vaccines is not influenced by lactation. Current recommendations state that vaccination should be considered for all individuals aged 6 months and older, including those who are pregnant or trying to conceive now or in the future and breastfeeding, irrespective of their previous COVID-19 infection status^23^. Our study contributes to the burden of evidence^24^ suggesting that there was no immediate harm for breastfeeding women receiving anti-SARS-CoV-2 vaccine or their breastfed infants. Also, seven study participants got pregnant after vaccination, one 2 weeks after the first dose, one 4 months after the first dose, and 5 over 12 months after the first dose. Recently conducted long-term follow-up interviews of study participants confirmed that there were also no unforeseen harms for breastfeeding women or their breastfed infants, or any other participant, two years after receiving the anti-SARS-CoV-2 vaccine.

The appearance of new variants of concern as well as the existence of reports of a fast decline in anti-SARS-CoV-2 antibody levels after natural infection and vaccination^25^ have raised uncertainty about the role of the components of cellular immunity. A few studies exploring the immunological response of vaccinated individuals in Serbia have reported only humoral immunity^19,20^. In this study, an increased expression of costimulatory receptors CD28 on CD4^+^ T cells and CD80 on B cells was detected 2 months after the administration of the second vaccine dose. Furthermore, the expression of CD80 on monocytes increased 1-month after complete vaccination. T cells proliferation and differentiation into effector or memory cells require antigen presentation, co-stimulation, and cytokines. The best-defined co-stimulators of T cells are pairs of molecules, B7-1 (CD80) and B7-2 (CD86). They are typically expressed on antigen-presenting cells (APCs) such as dendritic cells, B cells, macrophages, monocytes and T-cells. Their expression on monocytes is also important for activating lymphocytes and adaptive immunity^26^. The two main receptors present on the surface of T lymphocytes that bind CD80 and CD86 are CD28, and the cytotoxic T-lymphocyte associated protein 4 (CTLA-4). Binding to CD28 activates lymphocytes and consequently enhances the immune response^27^. The results obtained in this study show that CD4^+^ T cells are activated by the S antigen derived epitopes presented on activated B cells and monocytes 2 months after the second dose of vaccine. Various data suggested that CD4 T cells were predominantly activated after mRNA vaccine^28,29^, while in some studies both CD4 and CD8 T cells are reported to be equally presented and activated^30^. Tan et al. recently observed that at 3 months after vaccination, the mean quantity of Spike-specific T cells was equivalent to what has been detected in convalescent patients at a similar time after SARS-CoV-2 infection^31^. Furthermore, the components of adaptive immunity (both in previously infected and vaccinated) appear to be independently regulated^6,32,33^ and there is a lack of correlation between antiviral antibody production and T cell frequency. In addition, current mRNA vaccines elicit immune responses confined to the Spike protein. However, the potentially protective role of T cells specific for other structural and nonstructural proteins will also have to be evaluated^34^. The idea that polyantigenic vaccines might exert greater control against viral variants than monoantigenic ones, like Spike protein-based vaccines, might have a sound rationale^35,36^.

In this study increased levels of IFN-γ were detected in all lymphocytes and in CD3^+^ T cells 7 days after complete vaccination. Also, an increased level of IFN-γ in serum of all vaccinated individuals was observed, both in SARS-CoV-2 naïve and previously infected individuals 7 days after full vaccination. Thus, the mRNA-based vaccine-induced Spike-specific T cells that abundantly produce IFN-γ. IFN-γ is a key cytokine with antiviral properties and has been shown to inhibit the replication of SARS-CoV-2^8^. The fast elicitation of IFN-γ by the BNT162b2 vaccine demonstrated in this study indicates a favorable T cell immune response against the SARS-CoV-2 virus.

The immunological reaction triggered by SARS-CoV-2 also includes the production of numerous proinflammatory cytokines, primarily IL-6 and TNF-α and changes in their levels might be associated with the prognosis of the disease. Severe and fatal cases of COVID-19 were reported in individuals with increased cytokine levels^37^. However, in this study, no significant difference in serum levels of IL-6 and TNF-α was detected 7 days and up to 1 month after full vaccination, which might result from specific cohort characteristics and timing of sampling.

These data represent first published reports on durable immunity taking into account both the humoral and the cellular component, that focuses on breastfeeding women. This might be especially useful for future meta analyses of this and other sensitive population subgroups, as data from the Balkan area on Slavic population is usually missing from larger multinational efforts.

## Conclusions

The anti-SARS-CoV-2 BNT162b2 mRNA vaccine-induced measurable and durable humoral and cellular responses in breastfeeding women and both naïve and previously infected individuals in the first six months after vaccination in Serbia. No serious adverse effects to vaccination were reported, and only 5% of participants were infected with the SARS-CoV-2 in the first six months after vaccination, without the need for hospitalization. No unforeseen harms for breastfeeding women or their breastfed infants were reported two years after receiving the anti-SARS-CoV-2 vaccine***.*** Next studies in the field should focus on vaccine research and development strategies that might keep the COVID-19 and potential similar pandemics under faster and long-lasting control. Efforts also need to be invested to provide knowledge-based guidelines, properly address misinformation especially surrounding sensitive subpopulations as breastfeeding women, facilitate access to vaccines at times of high need and reinforce general trust in science.

## Ethics approval

The procedures in this study were approved by the Ethics Committee of the Institute for Oncology and Radiology of Serbia (No. 899–01 from 01.04.2021.) and were in accordance with the Helsinki Declaration of 1975, as revised in 2013. All participants were interviewed by experienced medical staff and signed an informed consent for participation and publication.

## Supplementary Information

Supplementary Information 1

Supplementary Information 2

## Data Availability

Data obtained from this study is confidential and may be obtained from the corresponding author upon reasonable request.
