# Supplementary figures and images for: Detection of humoral and cellular immune response to anti-SARS-CoV-2 BNT162b2 vaccine in breastfeeding women and naïve and previously infected individuals
Source: Sci Rep. 2023 Apr 17;13:6271. doi: 10.1038/s41598-023-33516-1 (PMC10109231; doi:10.1038/s41598-023-33516-1)

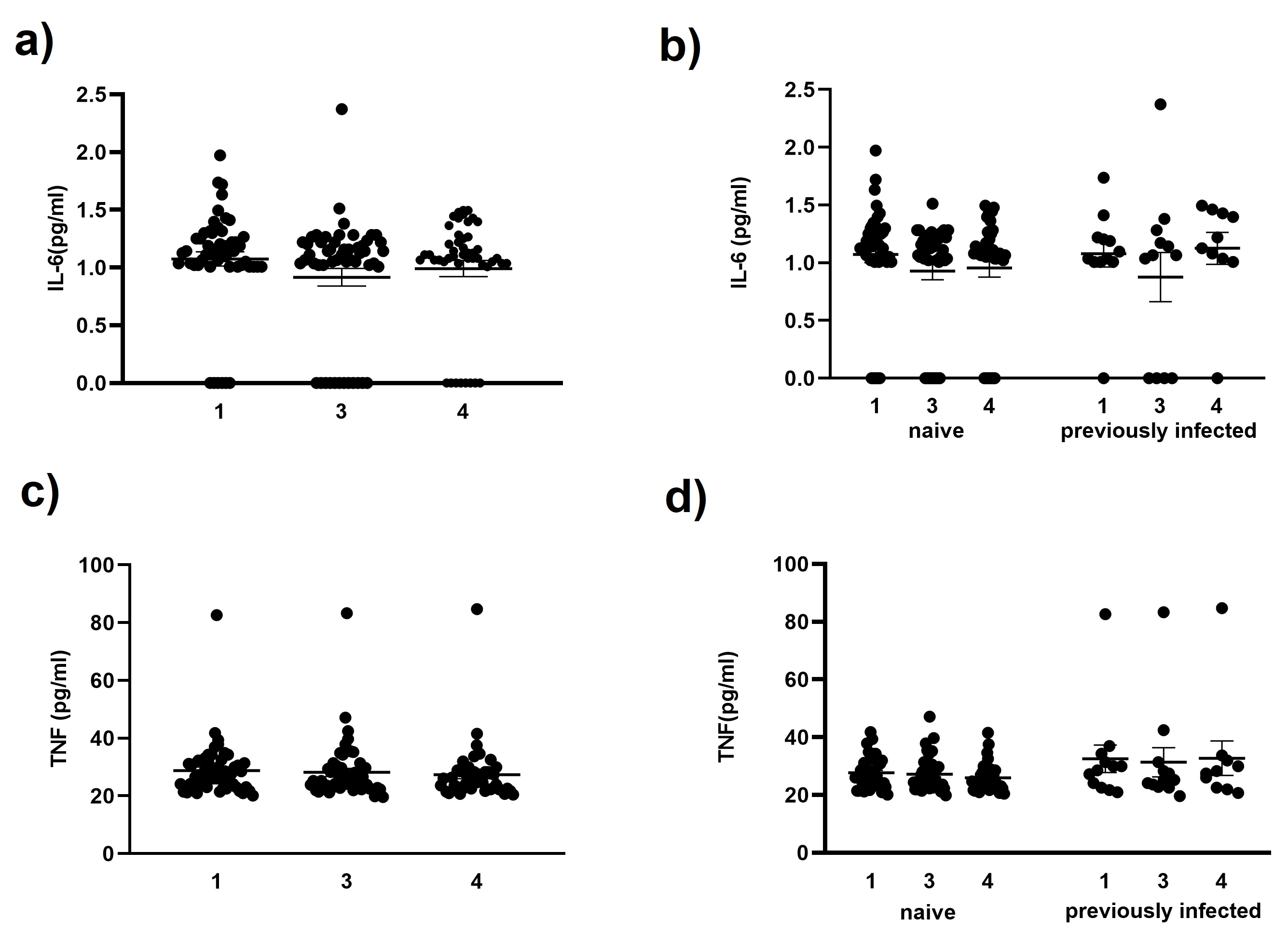

Supplement: Supplementary file 1 — Supplementary Information 1 [file 41598_2023_33516_MOESM1_ESM.jpg]
