# Supplementary material for: Detection of humoral and cellular immune response to anti-SARS-CoV-2 BNT162b2 vaccine in breastfeeding women and naïve and previously infected individuals
Source: Sci Rep. 2023 Apr 17;13:6271. doi: 10.1038/s41598-023-33516-1 (PMC10109231; doi:10.1038/s41598-023-33516-1)
Supplement: Supplementary file 2 — Supplementary Information 2 [file 41598_2023_33516_MOESM2_ESM.docx]

**Supplementary Figure 1.** Serum levels of IL-6 in the (a) whole analyzed group, (b) naïve and previously infected individuals; serum levels of TNF in the (c) whole analyzed group, (d) naïve and previously infected individuals. The results are presented as individual values with mean ± standard error of the mean (SEM).
